# Diseases of Children

**Published:** 1879-08

**Authors:** 


### DISEASES OF CHILDREN.

No infant or child of good constitution, who has the comforts of a home and *proper medicine*, should be allowed to die of *cholera infantum or other bowel troubles incident to teething children.* The seat of these troubles is in the *liver*, which does not properly perform its functions. Everything that the child swallows passes through its body unchanged, and consequently affords no nourishment to the system, but irritates and inflames the mucous lining of the stomach and intestines, causing pain, which is manifested in plaintive moans.

Under these accumulated troubles it sinks rapidly, and dies, literally, of starvation. The end is usually hastened by the well-meaning, but unfortunate administration of "soothing syrup," or some other preparation of opium. This checks the discharges, and the little patient, stupified by the drug, is quiet and seems better; but these appearances are too often delusive. The fluids accumulate in the stomach and bowels, and when these are full, finding no other escape, a portion are effused into the lungs and brain, and the child perishes of congestion. This is the history of nearly every fatal case, and a history that will repeat itself until physicians substitute something else for the opium treatment, which the majority cling to with such tenacity. Any change would be for the better. It is just as safe to arrest with opium the discharges of a child who is suffering from "summer complaint," as it is to screw down the safety-valve of a steam boiler, and not a whit more so.

Why reputable physicians should employ opium in the treatment of these diseases, or any other diseases, of children, is to me a mystery "past finding out." Will this cruel and utterly useless slaughter of the innocents never cease?

It is a mistake to suppose that these diseases exist only in cities. They are caused by the irritation of teething, excessively warm weather, improper food, bad air, etc., etc. These causes exist everywhere, though in the country there are counteracting influences, which modify the disease. The complaint generally affects children between the ages of three months and two years, though instances occur before and after these periods.

